# Hepatitis B Infection in Outpatients and Pregnant Women Visiting a Mission Hospital in Ghana

**DOI:** 10.1002/puh2.70071

**Published:** 2025-06-14

**Authors:** Margaret Addo, Sule Apaame, Michael Abbey Ghanney, Hannah Konadu Adu, Michael E. DeWitt, Seth Offei Addo

**Affiliations:** ^1^ Gbawe Seventh Day Adventist Hospital Gbawe Ghana; ^2^ Department of Medical Microbiology University of Ghana Medical School, University of Ghana Accra Ghana; ^3^ Department of Internal Medicine Section on Infectious Diseases Wake Forest University School of Medicine Winston‐Salem North Carolina USA; ^4^ Center for the Study of Microbial Ecology and Emerging Diseases Wake Forest University School of Medicine Winston‐Salem North Carolina USA; ^5^ Department of Biology Wake Forest University Winston‐Salem North Carolina USA; ^6^ Parasitology Department Noguchi Memorial Institute for Medical Research, College of Health Sciences, University of Ghana Accra Ghana

**Keywords:** Ghana | hepatitis B virus (HBV) infection | outpatients | pregnant women

## Abstract

Millions of individuals worldwide suffer from hepatitis B, a serious, potentially fatal liver infection brought on by the hepatitis B virus (HBV). Although vaccines are available for HBV, infections continue to persist in Ghana. This study reports the prevalence of HBV infections in outpatients and pregnant women attending antenatal care at the Seventh‐day Adventist (SDA) Hospital in Gbawe, Ghana. This retrospective cohort study involved the review of de‐identified medical records of outpatients and pregnant women who visited the hospital between 2022 and 2024. Data on their HBV infection status, sex and age were analysed using R version 4.4.1. A total of 531 outpatients and 768 pregnant women visited the hospital during the study period. The prevalence of HBV infection was 7.5% in outpatients and 3.9% in pregnant women. It was observed that outpatients were more likely to be hepatitis B surface antigen (HBsAg) positive (OR = 2.0, 95%CI = 1.24–3.28, *p* = 0.005). It was also seen that HBV prevalence increased from 2022 to 2023 and decreased in 2024. There is a need for more educational campaigns to raise awareness of HBV infections, especially in pregnant women due to the risk of mother‐to‐child transmission. Furthermore, vaccinations need to be made affordable and easily accessible to the general population to ensure maximum coverage within the country.

## Graphical abstract

• Hepatitis B infection was detected in outpatients and pregnant women.

• Infections in pregnant women suggest a risk of mother‐to‐child transmission.

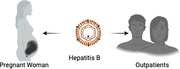

## 1 Introduction

According to the World Health Organization, there are an estimated 254 million individuals living with chronic hepatitis B virus (HBV) infection globally, making it a major global health concern [1]. Acute and chronic liver diseases, such as cirrhosis and hepatocellular carcinoma, are caused by the virus, which is mainly spread via contact with infectious bodily fluids [2]. In sub‐Saharan Africa, the incidence rate of HBV is 6%–10% [3]. The high incidence of hepatitis B in many low‐ and middle‐income nations makes efficient screening and preventative measures necessary to lower transmission rates. Serological indicators are typically used for HBV screening, and the main marker for detecting an active infection is hepatitis B surface antigen (HBsAg) [4].

It has been reported that 10%–15% of Ghana's population is infected with HBV, making it one of the worst‐affected nations in the world [5]. According to estimates, Ghana's overall HBV‐related death rate has increased by 17.2% over the last two decades, mostly due to a lack of voluntary testing for early detection and treatment [5]. This means that more efforts need to be put in place to encourage HBV testing amongst the general population.

Antenatal clinics (ANCs) and outpatient departments (OPDs) are crucial locations for HBV screening since they come with advantages such as early diagnosis, treatment or vaccination and essential education on preventive measures [6]. In expectant mothers, HBV can cause chronic infection in the foetus and significantly increase the total burden of HBV [7]. As such, there is a need for antenatal screening and prompt intervention to prevent infection spread [8, 9].

Comprehensive screening efforts are frequently hampered in areas with limited resources due to stigma, a lack of awareness, and a lack of proper healthcare infrastructure [5]. To improve patient outcomes and inform public health efforts, it is imperative to comprehend the prevalence and distribution of HBV in outpatients and pregnant women. This study examined the medical records of patients visiting the antenatal and outpatient units of the Gbawe Seventh‐day Adventist (SDA) hospital to determine the prevalence of HBV infection. In addition to helping direct targeted initiatives to lower transmission and enhance health outcomes, the findings will offer important insights into the burden of HBV in these groups.

## 2 Methods

The healthcare system in Ghana is a hybrid public‐private model that is designed to offer services at various clinical and administrative levels while continuously working to increase equity, quality and accessibility. The system's structure and organization are based on three primary administrative levels: district, regional and national. The Gbawe SDA hospital is a secondary‐level healthcare facility. The hospital, located in an urban setting, serves the inhabitants of Mallam–Gbawe and its environs within the Greater Accra Region. With a 32‐bed capacity, it provides a variety of services, including an OPD, a theatre, admission wards, a laboratory, emergency and a pharmacy department.

In this study, the medical records of outpatients and pregnant women who visited the Gbawe SDA hospital from May 2022 to September 2024 were reviewed. Only those who were tested for HBV during the study period were included in the study. Data on their HBV infection status, sex and age were retrieved and analysed. The patients were screened for HBV using HBsAg rapid diagnostic kits according to the manufacturer's instructions.

The test was performed as follows: after taking the test cassette out of the sealed pouch, it was set with the sample well up on a spotless, flat surface. Three drops of whole blood, serum or plasma were vertically dropped into the sample well using the disposable plastic straw. In the case of whole blood, a drop of sample diluent was added to the sample well. The test result was observed within 15–20 min.

Ethical approval for this study was obtained from the Ghana Adventist Health Services Ethics Review Committee (GAHS/ERC/040/25).

### 2.1 Statistical Analysis

We described the study cohort baseline characteristics and testing positivity by outpatient versus pregnant women using descriptive statistics including medians and interquartile ranges for continuous variables and counts and proportions for categorical variables. Differences between the two groups were assessed using Wilcoxon rank sum tests (continuous) and Chi‐square tests (categorical). To assess factors associated with testing positive for HBV, we conducted univariate logistic regression. We then plotted positive over time overall and stratified by patient status and sex to visually assess trends. All analysis was conducted using R version 4.4.1.

## 3 Results

A total of 1299 patients visited the hospital, with 531 (40.9%) recorded as outpatients and 768 (59.1%) as pregnant women (Table 1). With the outpatients, the ages ranged from 14 to 77 years, with males representing 291 (55%) compared to females, who were 240 (45%). The pregnant women had ages ranging from 14 to 46 years. The yearly HBsAg positives were 15 (4.6%) in 2022, 40 (6.8%) in 2023 and 15 (3.9%) in 2024. The overall prevalence of HBV infection amongst the outpatients was 7.5% (*n* = 40), with the yearly prevalence recorded as 7.6% (*n* = 9) in 2022, 8.4% (*n* = 22) in 2023 and 5.9% (*n* = 9) in 2024. An overall prevalence of 3.9% (*n* = 30) was recorded for pregnant women, with the yearly prevalence as 2.8% (*n* = 6) in 2022, 5.5% (*n* = 18) in 2023 and 2.6% (*n* = 6) in 2024. The outpatients were more likely to test positive for HBV (OR = 2.0, 95%CI = 1.24–3.28, *p* = 0.005). Age, sex or year of testing was not associated with testing positive for HBV (Table 2).

**TABLE 1:** Summary of hepatitis B cases by case type.

Characteristic	Antenatal clinic, *N* = 768	Outpatient department, *N* = 531	*p* value^a^
**Age, median (IQR)**	30 (26–33)	37 (30–45)	<0.001
**Sex, *n* (%)**			<0.001
Female	768 (100)	291 (55)	
Male	0 (0)	240 (45)	
**Hepatitis B test result, *n* (%)**			0.004
Negative	738 (96)	491 (92)	
Positive	30 (3.9)	40 (7.5)	
**Year, *n* (%)**			0.040
2022	211 (27)	118 (22)	
2023	328 (43)	261 (49)	
2024	229 (30)	152 (29)	

^a^ Wilcoxon rank sum test; Pearson's Chi‐squared test.

**TABLE 2:** Logistic regression for factors associated with hepatitis B positivity.

Characteristic	*N*	OR (95%CI)	*p* value
**Clinic type**	1299		
Antenatal clinic		—	
Outpatient department		2.00 (1.24–3.28)	0.005
**Year**	1299	0.93 (0.67–1.28)	0.64
**Age**	1299	1.01 (0.98–1.03)	0.52
**Sex**	1299		
Female		—	
Male		1.22 (0.65–2.14)	0.51

Abbreviations: CI, confidence interval; OR, odds ratio.

### 3.1 Trends of HBV Positivity

**FIGURE 1:**
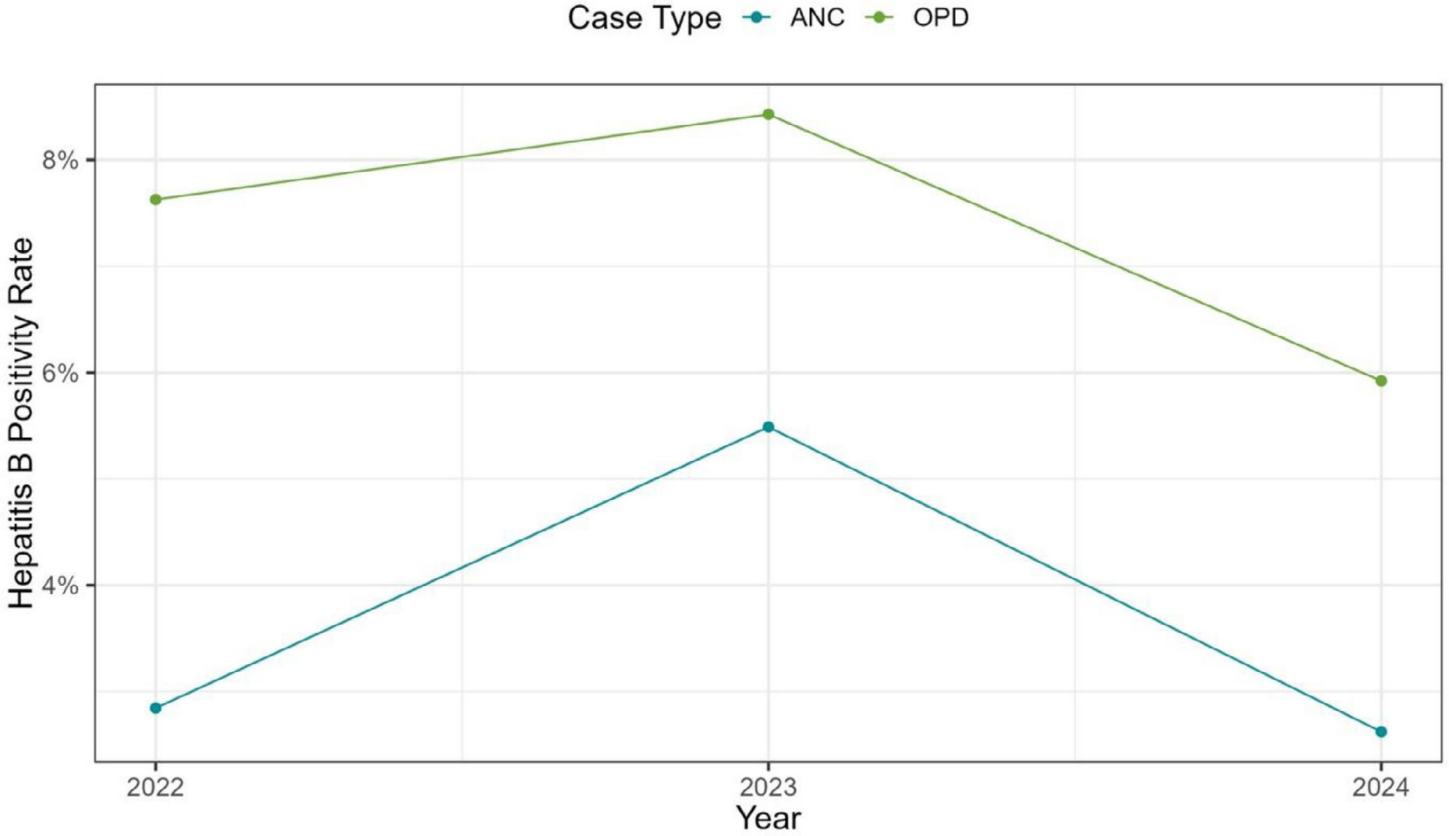
Trends of HBV prevalence in outpatients and pregnant women. ANC, antenatal clinic, OPD, outpatient department.

**FIGURE 2:**
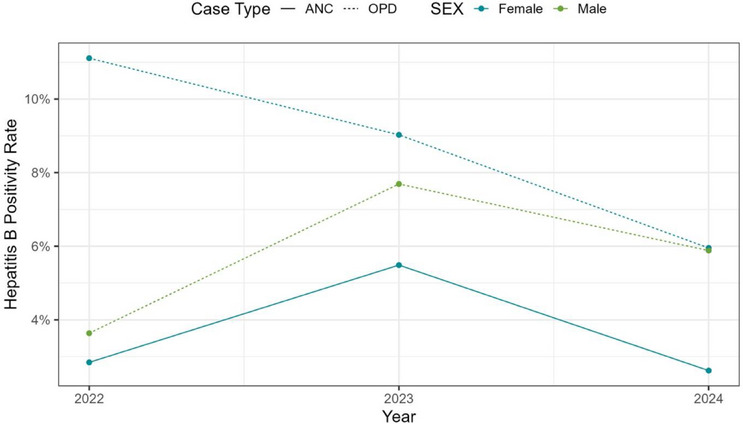
Trends of HBV prevalence based on the sex of patients. ANC, antenatal clinic, OPD, outpatient department.

It was observed in both outpatients and pregnant women that there was an increase in HBV prevalence from 2022 to 2023 and a decrease in 2024 (Figure 1). Considering the sex of the patients, it was observed that with the pregnant women, there was an increase in HBV positivity from 2022 to 2023 and a decrease in 2024. With the outpatients, although HBV positivity decreased in females from 2022 to 2024, there was an increase from 2022 to 2023 in males with a decrease in 2024 (Figure 2).

## 4 Discussion

This study provides important insight into the prevalence of HBV infection among pregnant women and outpatients visiting the Gbawe SDA hospital. The combined prevalence of 5.4% indicates a significant burden and a need for ongoing surveillance and targeted public health interventions.

The potential of vertical transmission makes the incidence of HBV in ANC patients very worrisome. About 70%–90% of babies born to mothers with HBV may develop a chronic infection if treatment is not received. This is a serious concern because of the increased risk of developing chronic liver disease [10]. Studies in Ghana have reported mother‐to‐child transmission of HBV to be 8.3% [11] and 5.9% [12], calling for an urgent need to prevent or reduce transmission. In this study, 3.9% of pregnant women who visited the hospital were HBsAg positive. This is higher than a previous study in Ghana which reported an HBV prevalence of 3.3% in pregnant women receiving antenatal care [13]. The prevalence of HBV recorded in this study is, however, lower than in other studies which reported prevalence rates of 9.5% [14] and 10.5% [15] in pregnant women. The varying prevalence rates could be due to the different study locations and sample sizes of the studies. The findings of our study support the WHO's recommendation that all pregnant women be checked for HBsAg to facilitate early intervention and highlight the significance of HBV screening as a crucial component of ANC services [16]. The observed prevalence of HBsAg among pregnant women underscores the need for preventive strategies against vertical transmission. It has been reported that in Ghana, some barriers to hepatitis B birth dose (HepB‐BD) vaccination are ignorance of mothers, high cost of vaccines and sometimes unavailability of the vaccines [17]. This can be resolved through the provision of free or less expensive vaccines at various health facilities and educating expectant mothers on the risk and transmission of HBV. Furthermore, the strengthening of HBV screening during routine ANC visits and ensuring follow‐up testing for infants born to HBV‐positive mothers could reduce the rate of vertical transmission.

Again, in this study, 7.5% of the outpatients were HBsAg positive. Despite ongoing vaccination initiatives, data from the outpatients indicates a sustained burden in the broader adult population, indicating that community‐based transmission persists. It may be possible to find asymptomatic carriers who unintentionally aid in community spread by routine screening in outpatients. By making HBV tests free and available at resource‐limited health facilities, a lot more outpatients can be screened, leading to early detection and management to avoid long‐term liver morbidity and mortality [18].

There are challenges when it comes to the fight against HBV infections in Ghana. A study showed a low level of awareness of HBV in certain regions of Ghana [19]. Furthermore, it was observed that people living with HBV had limited knowledge of the effects of the infection, and it harmed their lives [20]. Some HBV‐infected individuals also face stigmatization due to the belief that the disease is contagious, severe and caused by curses [21]. Another study also showed that people living with chronic HBV face some level of discrimination [22]. To reduce the spread of HBV, it is crucial to employ a variety of strategies, such as educational programmes to target populations and create awareness in communities, training of health workers and the encouragement of HBV vaccinations [21]. In Ghana, targeted health promotion campaigns that leverage social media and local health workers’ influence may effectively reach diverse populations, promoting preventative behaviours [23, 24].

Our findings advocate for expanded HBV screening policies that encompass both ANC and general OPD patients. Given the prevalence of infections in our study population, a more inclusive screening strategy could enhance early detection and reduce the HBV burden in the community. A multi‐pronged approach involving universal infant immunization, vaccination and routine screening in adults would strengthen HBV control efforts in Ghana [25, 26]. Moreover, as part of a larger public health initiative, collaborations with international health organizations could support resource‐limited facilities in maintaining a steady supply of screening kits and vaccines.

## 5 Conclusion

This study provides critical insights into HBV prevalence in OPD and ANC populations, emphasizing the need for comprehensive screening, vaccination and preventive strategies. The continued HBV transmission observed over the study period underscores the urgency of addressing this public health challenge. By implementing universal screening policies and improving access to vaccination and antiviral treatments, significant strides can be made toward HBV elimination. The findings advocate for the integration of HBV screening within routine healthcare services and highlight the importance of maternal screening in preventing mother‐to‐child transmission.

## Author Contributions

**Margaret Addo**: investigation, data curation, writing – review and editing, methodology, conceptualization. **Sule Apaame**: investigation, methodology, writing – review and editing, data curation. **Michael Abbey Ghanney**: investigation, methodology, writing – review and editing. **Hannah Konadu Adu**: investigation, methodology, writing – review and editing. **Michael E. DeWitt**: investigation, methodology, formal analysis, writing – review and editing. **Seth Offei Addo**: conceptualization, investigation, writing – original draft, data curation, supervision.

## Conflicts of Interest

The authors declare no conflicts of interest.

## Data Availability

All the data supporting this study are included in the article.

## References

[1] WHO . Hepatitis B. in WHO (World Health Organization, 2024), https://www.who.int/news‐room/fact‐sheets/detail/hepatitis‐b.

[2] B. Custer , S. D. Sullivan , T. K. Hazlet , et al., “Global Epidemiology of Hepatitis B Virus,” Journal of Clinical Gastroenterology 38, no. 38 (2004): S158–S168, 10.1097/00004836-200411003-00008. 15602165

[3] A. Schweitzer , J. Horn , R. T. Mikolajczyk , G. Krause , and J. J. Ott , “Estimations of Worldwide Prevalence of Chronic Hepatitis B Virus Infection: A Systematic Review of Data Published Between 1965 and 2013,” Lancet 386 (2015): 1546–1555, 10.1016/S0140-6736(15)61412-X. 26231459

[4] R. G. Gish , B. D. Given , C. L. Lai , et al., “Chronic Hepatitis B: Virology, Natural History, Current Management and a Glimpse at Future Opportunities,” Antiviral Research 121 (2015): 47–58, 10.1016/j.antiviral.2015.06.008. 26092643

[5] R. Ofori‐Asenso and A. A. Agyeman , “Hepatitis B in Ghana: A Systematic Review & Meta‐Analysis of Prevalence Studies (1995–2015),” BMC Infectious Diseases 16, no. 1 (2016): 130, 10.1186/s12879-016-1467-5. 26987556 PMC4797341

[6] S. Sarah , C. Vellozzi , A. Reingold , et al., “Prevention of Hepatitis B Virus Infection in the United States: Recommendations of the Advisory Committee on Immunization Practices,” MMWR Recommendations and Reports 67, no. 1 (2018): 1–31, 10.15585/mmwr.rr6701a1. PMC5837403 29939980

[7] N. A. Terrault , A. S. F. Lok , B. J. McMahon , et al., “Update on Prevention, Diagnosis, and Treatment of Chronic Hepatitis B: AASLD 2018 Hepatitis B Guidance,” Hepatology 67, no. 4 (2018): 1560–1599, 10.1002/hep.29800. 29405329 PMC5975958

[8] C. Agulebe , M. Maanongun , L. Nombur , and P. Abu , “Prevalence of Hepatitis B Virus Infection Among Antenatal Care Attendee in Makurdi, Benue State, Nigeria: A 6‐Year Review of Tertiary Hospital Records,” Asian Research Journal of Gynaecology and Obstetrics 7, no. 1 (2024): 207–218.

[9] S. Thahir , E. Muhindo , B. Turigye , et al., “Implementation of Hepatitis B Screening Into Routine Antenatal Care to Prevent Mother‐to‐Child Transmission in Rural Western Uganda,” Open Forum Infectious Diseases 10, no. 9 (2023): ofad452, 10.1093/ofid/ofad452. 37705692 PMC10496864

[10] M. Belopolskaya , V. Avrutin , O. Kalinina , A. Dmitriev , and D. Gusev , “Chronic Hepatitis B in Pregnant Women: Current Trends and Approaches,” World Journal of Gastroenterology 27, no. 23 (2021): 3279–3289, 10.3748/wjg.v27.i23.3279. 34163111 PMC8218362

[11] D. Candotti , K. Danso , and J. P. Allain , “Maternofetal Transmission of Hepatitis B Virus Genotype E in Ghana, West Africa,” Journal of General Virology 88, no. 10 (2007): 2686–2695, 10.1099/vir.0.83102-0. 17872520

[12] T. Hambridge , Y. Nartey , A. Duah , and A. Plymoth , “Hepatitis B Mother‐to‐Child Transmission in the Eastern Region of Ghana: A Cross‐Sectional Pilot Study,” Pan African Medical Journal 33 (2019): 218, 10.11604/pamj.2019.33.218.17242. 31692718 PMC6814340

[13] P. K. Kwadzokpui , E. E. Akorsu , A. Abaka‐Yawson , S. S. Quarshie , S. A. Amankwah , and P. A. Tawiah , “Prevalence and Knowledge of Hepatitis B Virus Infection Among Pregnant Women in the Ningo‐Prampram District, Ghana,” International Journal of Hepatology 2020 (2020): 7965146, 10.1155/2020/7965146. 32411482 PMC7204257

[14] R. Ephraim , I. Donko , S. A. Sakyi , J. Ampong , and H. Agbodjakey , “Seroprevalence and Risk Factors of Hepatitis B and Hepatitis C Infections Among Pregnant Women in the Asante Akim North Municipality of the Ashanti Region, Ghana; a Cross Sectional Study,” African Health Sciences 15, no. 3 (2015): 709–713, 10.4314/ahs.v15i3.2. 26957956 PMC4765455

[15] Y. Cho , G. Bonsu , A. Akoto‐Ampaw , et al., “The Prevalence and Risk Factors for Hepatitis B Surface Ag Positivity in Pregnant Women in Eastern Region of Ghana,” Gut and Liver 6, no. 2 (2012): 235–240, 10.5009/gnl.2012.6.2.235. 22570754 PMC3343163

[16] WHO . Prevention of Mother‐To‐Child Transmission of Hepatitis B Virus: Guidelines on Antiviral Prophylaxis in Pregnancy (WHO, 2020). 32833415

[17] C. A. Adjei , D. Suglo , A. Y. Ahenkorah , S. E. MacDonald , and S. Richter , “Barriers to Timely Administration of Hepatitis B Birth Dose Vaccine to Neonates of Mothers With Hepatitis B in Ghana: Midwives' Perspectives,” SAGE Open Nursing 9 (2023): 23779608231177547, 10.1177/23779608231177547. 37261100 PMC10227873

[18] S. C. Nwokediuko , “Chronic Hepatitis B: Management Challenges in Resource‐Poor Countries,” Hepatitis Monthly 11, no. 10 (2011): 786–793, 10.5812/kowsar.1735143X.757. 22224076 PMC3234575

[19] P. Mkandawire , C. Richmond , J. Dixon , I. N. Luginaah , and J. Tobias , “Hepatitis B in Ghana's Upper West Region: A Hidden Epidemic in Need of National Policy Attention,” Health & Place 23 (2013): 89–96, 10.1016/j.healthplace.2013.06.001. 23811012

[20] C. A. Adjei , F. Naab , and E. S. Donkor , “Beyond the Diagnosis: A Qualitative Exploration of the Experiences of Persons With Hepatitis B in the Accra Metropolis, Ghana,” BMJ Open 7, no. 11 (2017): e017665, 10.1136/bmjopen-2017-017665. PMC5722085 29102991

[21] C. A. Adjei , S. E. Stutterheim , F. Naab , and R. A. C. Ruiter , “Chronic Hepatitis B Stigma in Ghana: A Qualitative Study With Patients and Providers,” BMJ Open 9, no. 6 (2019): e025503, 10.1136/bmjopen-2018-025503. PMC6597648 31248915

[22] C. Freeland , A. Qureshi , J. Wallace , et al., “Hepatitis B Discrimination: Global Responses Requiring Global Data,” BMC Public Health [Electronic Resource] 24, no. 1 (2024): 1575, 10.1186/s12889-024-18918-8. 38862929 PMC11167835

[23] C. A. Adjei , S. E. Stutterheim , F. Bram , F. Naab , and R. A. C. Ruiter , “Correlates of Hepatitis B Testing in Ghana: The Role of Knowledge, Stigma Endorsement and Knowing Someone With Hepatitis B,” Health and Social Care in the Community 30, no. 6 (2022): e4564–e4573, 10.1111/hsc.13860. 35701984 PMC10083906

[24] K. A. Kusi , W. van der Puije , D. A. Asandem , et al., “World Hepatitis Day 2021—Screening and Vaccination Against Hepatitis B Virus in Accra, Ghana,” BMC Public Health [Electronic Resource] 23, no. 1 (2023): 1164, 10.1186/s12889-023-16108-6. 37328849 PMC10273713

[25] V. Papastergiou , R. Lombardi , D. MacDonald , and E. A. Tsochatzis , “Global Epidemiology of Hepatitis B Virus (HBV) Infection,” Current Hepatitis Reports 14, no. 3 (2015): 171–178, 10.1007/s11901-015-0269-3.

[26] W. Walana , M. Al‐Azab , I. B. Yabasin , and A. Abdul‐Mumin , “Childhood Immunization in Ghana: Tracing the History and Projecting the Future,” Public Health Challenges 3, no. 2 (2024): e176, 10.1002/puh2.176.

